# Hypoxia Enhances Activity and Malignant Behaviors of Colorectal Cancer Cells through the STAT3/MicroRNA-19a/PTEN/PI3K/AKT Axis

**DOI:** 10.1155/2021/4132488

**Published:** 2021-11-09

**Authors:** Yingchun Tang, Xiahui Weng, Chang Liu, Xing Li, Chao Chen

**Affiliations:** ^1^Department of Anorectal Surgery, The Eighth Hospital of Wuhan, Wuhan, 430010 Hubei, China; ^2^Department of Nursing, The Eighth Hospital of Wuhan, Wuhan, 430010 Hubei, China

## Abstract

Hypoxia is a typical microenvironment feature in almost all solid tumors and is frequently associated with growth of cancers including colorectal cancer (CRC). This study focuses on the influence of hypoxic microenvironment on the activity of CRC cells and the molecules involved. CRC cells were cultured under hypoxic conditions for 48 h, after which the proliferation, migration, invasion, and epithelial-mesenchymal transition activities of cells were increased. MicroRNA- (miR-) 19a was significantly upregulated in cells after hypoxia exposure according to a microarray analysis. STAT3 was confirmed as an upstream regulator of miR-19a which bound to the promoter region of miR-19a at the 96 bp/78 bp sites, and miR-19a bound to the PTEN mRNA to activate the PI3K/AKT signaling pathway. Hypoxia exposure induced STAT3 phosphorylation and PTEN knockdown in CRC cells. Silencing of STAT3 reduced the hypoxia-induced activity of CRC cells, whereas the malignant behaviors of cells were restored after miR-19a upregulation but blocked after PTEN overexpression. Similar results were reproduced *in vivo* where downregulation of STAT3 or overexpression of PTEN suppressed tumor growth and metastasis in nude mice. This study demonstrated that hypoxia augments activity and malignant behaviors of colorectal cancer cells through the STAT3/miR-19a/PTEN/PI3K/AKT axis.

## 1. Introduction

Colorectal cancer (CRC) is a prevalent malignancy worldwide, and the mortality caused by it in China has exceeded the world average by 17%, which has not seen a significant decline in the past years [1]. Approximately 20% of all patients are found at advanced stages at the initial diagnoses owing to the lack of early signs, and the relapse and mortality rates of these patients were significantly higher than those with localized disease after colectomy [2]. In addition to late diagnosis, the limitation in the understanding of mechanisms underlying cancer recurrence and metastasis represents a major obstacle in cancer management.

One well-established characteristic of tumors is the rapid and uncontrolled proliferation, which limits the availability of oxygen, and hypoxia is a typical microenvironment feature in almost all solid tumors [3]. Limited oxygenation (hypoxia) is frequently associated with cancer growth and treating inefficacy [4]. Under hypoxic conditions, glycolysis maintains survival of cancer cells and enhances cell progresses, including proliferation, migration, and invasion [5]. An increased concentration of hypoxia-inducible transcription factors (HIFs) is usually involved, which is correlated with poor prognoses in many cancers, including CRC [6]. Identifying molecules involved in cancer progression, especially under hypoxic microenvironments, may help develop more ideas for CRC control.

MicroRNAs (miRNAs) are a class of short noncoding RNAs which fulfill potent functions in fundamental cellular processes such as proliferation and differentiation through the regulation of target mRNA transcripts [7]. miRNAs can work as either oncogenes or tumor suppressors whose dysregulation has been frequently observed in the carcinogenesis of CRC through the regulation of critical mRNAs and signaling pathways [8]. Importantly, emerging evidence has suggested that miRNAs may be affected by tumor microenvironments, including hypoxia, and they play crucial roles in the responses induced by hypoxia [9]. In this work, a miRNA microarray analysis identified highmiR-19a expression in CRC cells after exposure to hypoxic conditions. miR-19a has been reported to enhance proliferation, aggressiveness, and lymphangiogenesis in CRC [10]. The relevance of hypoxia microenvironment to miR-19a expression and the mechanism remain unknown, and the function of miR-19a in CRC progression yet requires further investigation.

Our integrated bioinformatic analyses and cellular experiments predicted that signal transducer and activator of transcription 3 (STAT3) possibly serves as an upstream regulator of miR-19a, whereas phosphatase and tensin homolog (PTEN) serves as an important target of miR-19a. STAT3 is a member of the STAT family comprising transcription activators and signal transducers that play key roles in multiple cellular functions such as proliferation and differentiation, and STAT3 inhibition has been suggested as a promising option for anticancer therapy [11, 12]. PTEN is a phosphatase which directly blocks the activation of oncogenic phosphatidyl inositol 3-kinase/protein kinase B/mammalian target of rapamycin (PI3K/AKT/mTOR) signaling pathway, thus serving as an important tumor suppressor [13]. Taken together, we hypothesized that hypoxia microenvironment possibly affects development of CRC cells through the alternations of a STAT3/miR-19a/PTEN axis.

## 2. Materials and Methods

### 2.1. Cell Culture and Transfection

CRC cell lines HCT116 and SW480 and a human embryonic kidney- (HEK-) 293T cell line were acquired from American Tissue Culture Collection (ATCC, Manassas, VA, USA). A normal colon cell line NCM460 was acquired from BeNa Culture Collection (BNCC, Henan, China). HEK-293T and NCM460 cells were cultured in Dulbecco's modified Eagle's medium (DMEM), HCT116 cells were cultured in McCoy's 5A medium, whereas SW480 cells were cultivated in Leibovitz's L-15 medium. All media were acquired from Thermo Fisher Scientific Inc. (Waltham, MA, USA) and filled with 10% fetal bovine serum (FBS). The cells were cultured at 37°C in a humidified atmosphere enriched with 5% CO_2_, while the hypoxia-conditioned cells were cultured in a Billups-Rothenberg chamber (Del Mar, CA, USA) pumped with 1% O_2_, 94% N_2_, and 5% CO_2_.

The miR-19a mimic and miR-19a control for cell transfection were purchased from Exiqon (Woburn, MA, USA). The cDNA of miR-19a was cloned to pMCS-CMV lentiviral vectors (GeneChem Co., Ltd., Shanghai, China). Small-interfering (si) RNA of STAT3 was acquired from Addgene (Cambridge, MA, USA), and the overexpression vector of PTEN (PTEN-OE) was acquired from Santa Cruz Biotechnology (Santa Cruz, CA, USA). The vectors were transfected into CRC cells using Lipofectamine 2000 (Thermo Fisher Scientific), and the stably transfected cells were screened using 4 *μ*g/mL puromycin for further use.

### 2.2. Cell Counting Kit-8 (CCK-8) Method

HCT116 and SW480 cells were detached in the mixture of 0.02% ethylene diamine tetraacetic acid and 0.25% trypsin to prepare cell suspension. The suspension was loaded in 96-well plates at 100 *μ*L per well. The cells were cultured at 37°C with 5% CO_2_. At the 24, 48, 72, and 96 h, respectively, each well was filled with 10 *μ*L CCK-8 reagent (TAKARA, Otsu, Shiga, Japan) for another 2 h of incubation. Thereafter, the optical density (OD) value was examined at 460 nm to evaluate cell viability.

### 2.3. Transwell Assays

Cell invasion and migration abilities were examined by Transwell assays. For the invasion assay, Matrigel (BD, Franklin Lakes, NJ, USA) was mixed with serum-free medium at 1 : 9 and then loaded on the bottom of the 8 *μ*m Transwell apical chambers (Corning, Kennebunk, ME, USA) for 2 h at 37°C. After that, 5 × 10^4^ CRC cells were sorted into the apical chambers, and the basolateral chambers were filled with 10% FBS-DMEM for inducer. After 24 h, the cells invaded into the lower membranes were fixed in methanol (Santa Cruz, CA, USA) for 30 min, stained with 0.1% crystal violet (Sangon Biotech, Shanghai, China) for 5 min, and then observed under an inverted microscope (Olympus Optical Co., Ltd, Tokyo, Japan) with 5 random fields included. Cell migration was examined in a similar manner except for precoating Matrigel on the apical chambers.

### 2.4. Immunofluorescence Staining

Stably transfected cells were fixed in 4% methanol for 5 min, penetrated in 0.1% Triton X-100 (Sangon Biotech), and incubated with anti-Vimentin (1 : 1,000, ab16700, Abcam Inc., Cambridge, MA, USA) and anti-E-cadherin (1 : 100, ab194982, Abcam) at 20°C for 1 h and then with fluorescein isothiocyanate-labeled secondary antibody (1 : 5,000, ab150088, Abcam) at 37°C for 1 h. The slides were then sealed with antifluorescence quenching Vectashield (Vector Laboratories Inc., Burlingame, CA, USA). The nuclei were counterstained with 4′, 6-diamidino-2-phenylindole (Solarbio Science & Technology Co., Ltd., Beijing, China). All cells were examined using a confocal imaging system Zeiss LSM 510 (Zeiss Inc, AG, Oberkochen, Germany).

### 2.5. miRNA Microarray Analysis

Total RNA from HCT116 and SW480 cells cultured under hypoxic or normoxic conditions was extracted using the TRIzol reagent (Thermo Fisher Scientific), and the miRNA part was further purified using a mirVana miRNA extraction kit (Ambion, Austin, TX, USA). The isolated miRNAs were labeled with Hy3 using a miRCURY microarray labeling kit (Exiqon) and then hybridized using a miRCURY LNA miRNA Array (v.8.0, Exiqon). The microarray data were collected using a GenePix 4000B scanner (Axon Instruments, Molecular Devices, San Jose, CA, USA) and analyzed using a GenePix Pro 6.0 software (Axon Instruments).

### 2.6. Collection of Clinical Samples

Sixty-one CRC patients who were admitted into the Eighth Hospital of Wuhan from January 2015 to June 2016 were enrolled in this study. The tumor tissues and the adjacent nontumor tissues (> 5 cm away from the lesion sites) were collected during surgery. All patients were diagnosed as having CRC through histopathology examination and free of a history of adjuvant treatment. This study was ratified by the Ethical Committee of the Eighth Hospital of Wuhan and conducted according to the *Declaration of Helsinki*. Written informed consent form was received from each respondent.

### 2.7. Reverse Transcription Quantitative Polymerase Chain Reaction (RT-qPCR)

Total RNA from tissue homogenate and cells was extracted using the TRIzol reagent again. The RNA sample was eluted in 30 nuclease-free water and then quantified and purified using a ND-2000 NanoDrop ultraviolet spectrophotometer (Thermo Fisher Scientific) according to the manufacturer's instructions. After that, 1 *μ*g RNA was reverse-transcribed into cDNA in a Light Cycler 480 real-time PCR system at a 20 *μ*L volume using a PrimeScript RT kit (TAKARA). Next, real-time qPCR was conducted using a SYBR® Premix Ex Taq™ II kit (TAKARA) to quantify the expression of miR-19a and STAT3 and PTEN mRNAs. The primers are listed in Table 1, in which U6 was used as the internal control for miR-19a, while GAPDH was used as the control for mRNAs. Relative gene expression was quantified using the 2^−*ΔΔ*Ct^ method.

### 2.8. Dual-Luciferase Reporter Gene Assay

The promoter region of miR-19a was fragmented from the distal side to near side and fused with the cDNA of the Firefly and Renilla luciferases in the GV354 vector (GeneChem). All the constructed vectors were identified by sequencing. HEK-293T cells were sorted in 24-well plates in a 37°C incubator with 5% CO_2_ at 5 × 10^4^ cells per well. The vectors above were delivered into cells and maintained at 37°C for 12 h. The activity of Firefly and Renilla luciferase was examined on a dual-luciferase reporter gene detection system (Promega, Madison, WI, USA). The PTEN 3′UTR containing the wild-type (WT) binding sequence with miR-19a and the corresponding mutant-type (MT) sequence were constructed and inserted into pGL3 promoter vectors to construct pGL3-PTEN-WT and pGL3-PTEN-MT luciferase reporter vectors. After that, the miR-19a mimic or control was cotransfected with 500 ng pGL3-PTEN-WT or pGL3-PTEN-MT vectors into 2 × 10^4^ cells, and 50 ng pRL-SV40 Renilla luciferase vector was cotransfected as well to examine the transfection efficacy. After 48 h, the relative activity of Firefly and Renilla luciferase was examined using the dual-luciferase reporter gene detection system again.

### 2.9. Chromatin Immunoprecipitation- (ChIP-) qPCR

A ChIP assay was performed using a SimpleChIP Plus Kit (Cell Signaling Technologies (CST), Beverly, MA, USA) according to the kit's instructions. In short, 2 × 10^6^ HEK-293T cells were cultured in 10 cm dishes. Cells were crosslinked using 1% methanol, and the reaction was terminated by glycine. The chromatin was detached in micrococcal nuclease. Anti-IgG (negative control (NC), mouse monoclonal IgG (CST) and anti-STAT3 (1 : 1,000, sc-8019, Santa Cruz Biotechnology) were reacted with the Protein G magnet beads to form protein-nucleic acid complexes (immunoprecipitates). The chromatin precipitates were eluted and purified, and the enrichment of STAT3 fragments was quantified using qPCR.

### 2.10. Western Blot Analysis

Cells were dissolved in 2× sodium dodecyl sulfate (SDS) protein sample buffer which contained 100 mM Tris-HCl (pH = 6.8), 200 mM DTT, 4% SDS, 0.4% bromophenol blue, and 20% glycerol. The protein concentration was determined using a Bradford Protein assay kit II (Bio-Rad Laboratories, Hercules, CA, USA). After that, an equal amount of protein sample (25 *μ*g) was separated on 10% SDS-polyacrylamide gel electrophoresis (Bio-Rad, Hercules, CA, USA) and transferred on polyvinylidene fluoride membranes (Thermo Fisher Scientific). After being blocked by 1% bovine serum albumin for 1 h, the membranes were incubated with the primary antibodies to *β*-catenin (1 : 1,000, ab22656, Abcam), p-AKT (1 : 500, 66444-1-Ig, Proteintech Group, Chicago, IL, USA), AKT (1 : 800, 60203-2-Ig, Proteintech Group), p-STAT3 (1 : 2,000, sc-8059, Santa Cruz Biotechnology), p-PI3K (1 : 800, ab28356, Abcam), E-cadherin (1 : 800, 33-4000, Thermo Fisher scientific), Vimentin (1 : 1,000, MA5-11883, Thermo Fisher scientific), and GAPDH (1 : 800, 60004-1-Ig, Proteintech Group) at 4°C overnight. After that, the membranes were further incubated with alkaline phosphatase-conjugated goat anti-mouse (1 : 4,000, ab205719, Abcam) at 20°C for 1 h. The protein bands were visualized using the Pierce ECL Western Blotting Substrate (Thermo Fisher Scientific), and the image density was analyzed using a gel densitometer (Bio-Rad).

### 2.11. Growth of Xenograft Tumors In Vivo

Stably transfected CRC cells (1 × 10^6^) were injected into the ventral side of male thymic nude mice (4-5 weeks old, 20 ± 2 g, Vital River Laboratory Animal Technology Co., Ltd., Beijing, China). After injection, the volume (*V*) of the xenograft tumor was evaluated once a week as follows: *V* = *W*^2^ × *L* × 0.5, where “*W*” indicates the width and “*L*” indicates the length. After four weeks, the mice were euthanized through an administration of 1% pentobarbital sodium (150 mg/kg, intraperitoneal injection), after which the tumors were taken out and weighed. All animal procedures were conducted according to the Guidelines for Animal Care and Use (National Institutes of Health, Bethesda, Maryland, USA). Significant measures were made to reduce the suffering of animals.

### 2.12. Metastasis of Tumors In Vivo

Another batch of nude mice were used for *in vivo* metastasis experiments. Briefly, 4 × 10^6^ CRC cells were injected into the mice through the tail veins, and these mice were euthanized on the 45^th^ day after injection. The murine liver tissues were extracted for hematoxylin and eosin (HE) staining (Beyotime Biotechnology Co., Ltd., Shanghai, China). The collected tissues were immobilized in 4% paraformaldehyde for 30-50 min, rinsed, dehydrated, cleared, embedded, and cut into sections. The paraffin-embedded sections were put on glass slides, oven-dried at 45°C, dewaxed, and rehydrated in alcohol. Thereafter, the sections were stained with hematoxylin (5 min) and eosin (3 min), dehydrated, cleared, sealed, and observed under a microscope (Olympus) to count the number of metastatic nodules in liver tissues.

### 2.13. Statistical Analysis

Statistical analysis was performed using the SPSS22.0 (IBM Corp. Armonk, NY, USA). Measurement data were collected from at least three independent experiments. Difference between were analyzed by the unpaired *t*-test (two groups) or one-way or two-way analysis of variance (multiple groups) followed by Tukey's post hoc test. Survival rate of patients was analyzed using the Kaplan-Meier analysis. Gene enrichment analysis was performed by Fisher's exact test. Correlations between variables were analyzed by Pearson's correlation analysis. The log rank test was used for poststatistical analysis. All data were presented as the mean ± standard deviation (SD). ^∗^*p* < 0.05 was considered to show statistical significance.

## 3. Results

### 3.1. Hypoxia Stimulates Activity of the CRC Cells

**Figure 1:**
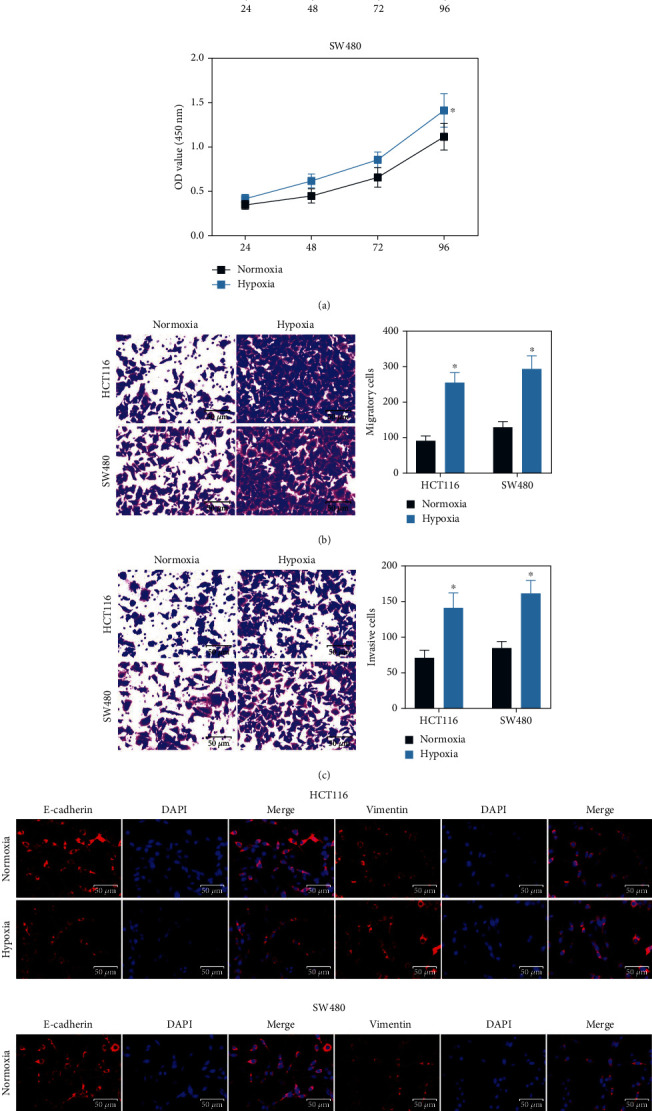
Hypoxia stimulates activity of the CRC cells: (a) viability of HCT116 and SW480 cells evaluated using the CCK-8 assay (^∗^*p* < 0.05, two-way ANOVA); (b, c) migratory (b) and invasive (c) activities of CRC cells examined by Transwell assays (^∗^*p* < 0.05, two-way ANOVA); (d) expression of EMT markers Vimentin and E-cadherin in cells determined by immunofluorescence staining. Data were presented as the mean ± SD from three independent experiments.

To confirm the influence of hypoxia on the activity of cells, HCT116 and SW480 cells were exposed to hypoxic conditions (1% CO_2_) for 48 h. After that, the CCK-8 assay suggested that compared to normal conditions (normoxia), hypoxia led to a notable increase in the viability of HCT116 and SW480 cells (Figure 1(a)). In addition, the number of cells that migrated or invaded into the lower membranes, according to the subsequent Transwell assays, was significantly increased after a hypoxia stimulation (Figures 1(b)–1(c)), indicating that hypoxia might also enhance the migratory and invasive potentials of cells. The expression of epithelial-mesenchymal transition- (EMT-) related markers Vimentin and E-cadherin was evaluated using immunofluorescence staining. It was found that the staining intensity of epithelial marker E-cadherin was decreased, whereas the intensity of the mesenchymal marker Vimentin was enhanced in cells exposed to hypoxic conditions (Figure 1(d)). Collectively, these cellular experiments confirmed that hypoxia might regulate the cell microenvironment and stimulate activity of the CRC cells.

### 3.2. miR-19a Is Upregulated in the Hypoxia-Exposed CRC Cells

**Figure 2:**
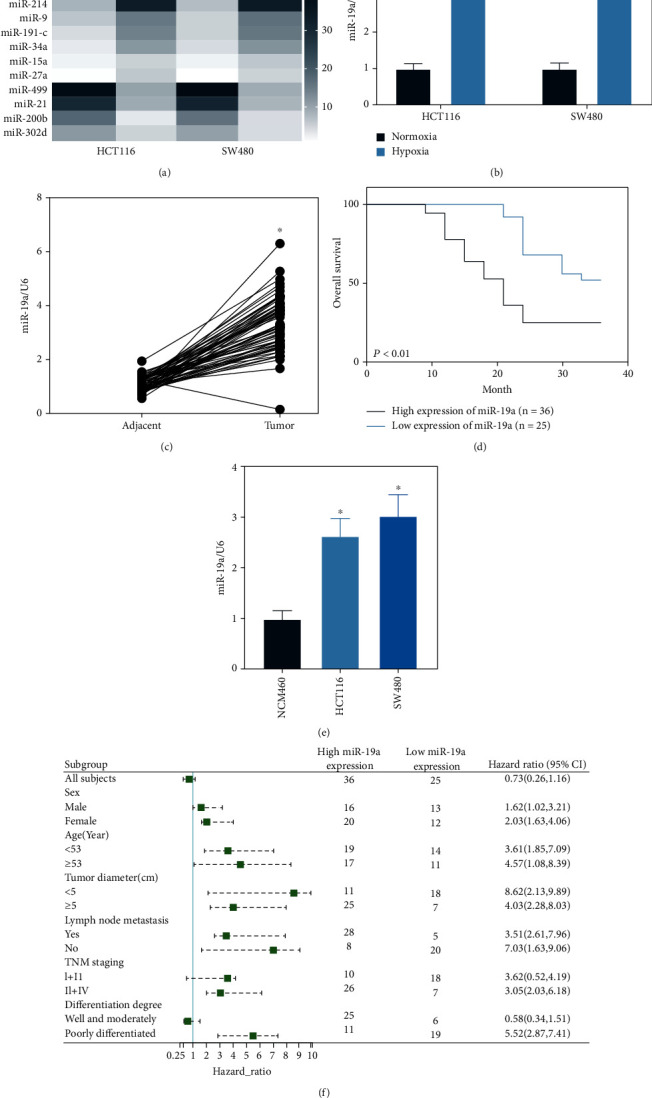
miR-19a is upregulated in the hypoxia-exposed CRC cells: (a) differentially expressed miRNAs between CRC cells cultured under hypoxic and normoxic conditions screened using a microarray analysis; (b) expression of miR-19a in HCT116 and SW480 cells cultured under hypoxic and normoxic conditions validated by RT-qPCR (^∗^*p* < 0.05, two-way ANOVA); (c) expression of miR-19a in cancer and paracancerous tissues from CRC patients examined by RT-qPCR (^∗^*p* < 0.05, paired *t*-test); (d) relevance of miR-19a expression to the survival of patients (^∗^*p* < 0.05, Kaplan-Meier analysis); (e) relevance of miR-19a expression to the clinical characteristics of patients; (f) expression of miR-19a in HCT116 and SW480 and in normal NCM460 cells determined by RT-qPCR (^∗^*p* < 0.05, one-way ANOVA). Data were presented as the mean ± SD from three independent experiments.

Following the findings above, we further explored the potentially involved molecules. A microarray analysis was conducted to identify the differentially expressed miRNAs between CRC cells cultured under hypoxic and normoxic conditions. It was noteworthy that the miR-19a was the most significantly upregulated miRNA in cells exposed to hypoxia (Figure 2(a)). This was validated by the subsequent RT-qPCR assay which showed that the expression of miR-19a in HCT116 and SW480 cells was upregulated after hypoxia exposure (Figure 2(b)). In the clinically collected tissues, miR-19a expression was found to be significantly upregulated in the cancer tissues compared to the paracancerous tissues (Figure 2(c)), and high expression of miR-19a was correlated with dismal prognosis of the patients (Figure 2(d)). The patients were allocated into high-miR-19a expression (*n* = 36) and low-miR-19a expression groups (*n* = 25) according to the mean value (3.95). The relevance of miR-19a expression to the clinical characteristics of patients was analyzed, and a forest graph (Figure 2(e)) was produced. It was found that high expression of miR-19a was correlated with increased tumor size, lymph node metastasis, TNM staging, and poor differentiation. In addition, the miR-19a expression in cancer cells and normal colon cells was examined. The RT-qPCR results showed that the expression of miR-19a was significantly elevated in HCT116 and SW480 cells relative to the NCM460 cells (Figure 2(f)). These results indicated that miR-19a was highly expressed in CRC and might be increased upon hypoxia stimulation.

### 3.3. STAT3 Activates Transcription Activity of miR-19a

**Figure 3:**
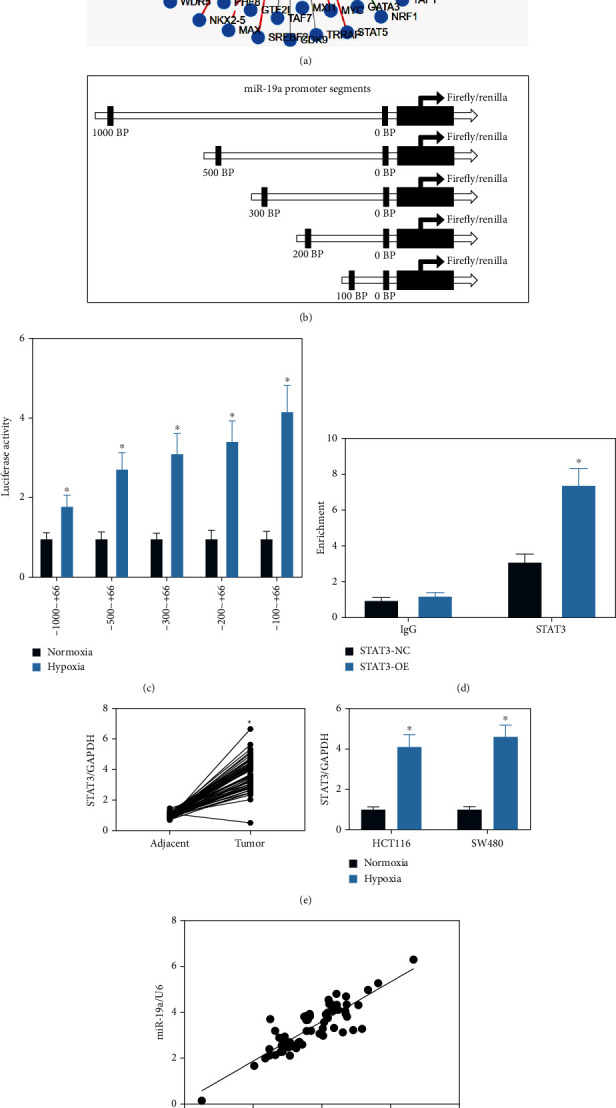
STAT3 activates transcription activity of miR-19a in the hypoxia-exposed CRC cells: (a) possible upstream regulators of miR-19a predicted using TransmiR v2.0; (b) five segments of miR-19a promoter divided from the distal side to near side; (c) luciferase activity of the miR-19a promoter segments in HEK293T cells after hypoxia exposure (^∗^*p* < 0.05, two-way ANOVA); (d) binding relationship between STAT3 and the miR-19a promoter validated using a ChIP-qPCR assay (^∗^*p* < 0.05, two-way ANOVA); (e) mRNA expression of STAT3 in tumor tissues and the paracancerous tissues examined by RT-qPCR; (f) a positive correlation between STAT3 and miR-19a in CRC tumor tissues (^∗^*p* < 0.05, Pearson's correlation analysis); (g) phosphorylation of STAT3 in HCT116 and SW480 cells after hypoxia exposure examined by western blot analysis. Data were presented as the mean ± SD from three independent experiments.

The possible regulators responsible for miR-19a upregulation in hypoxic microenvironment were explored. The upstream transcription factors of miR-19a were predicted using TransmiR v2.0 (http://www.cuilab.cn/transmir), and a number of transcription factors were suggested to have binding sites with the miR-19a promoter sequence (Figure 3(a)). The miR-19a promoter was obtained from the National Center for Biotechnology Information (NCBI) database (http://www.ncbi.nlm.nih.gov/pubmed/). Therefore, the miR-19a promoter was divided into five segments from the distal side to the near side, which were fused with the cDNA of the Firefly and Renilla luciferase (Figure 3(b)). Vectors containing different promoter sequence fragments were transiently transfected into HEK-293T cells, and the luciferase activity was examined using the dual-luciferase reporter gene system. The HEK-293T cells were cultured under normoxic and hypoxic conditions. It was found that hypoxia enhanced the transcription activity of miR-19a at all segments, whereas the greatest change appeared at the 100-66 bp segment (Figure 3(c)), indicating that the core regulation of miR-19a transcription under hypoxic conditions may occur at the 100 bp site. Among the candidate transcription factors, only STAT3 showed a binding site with miR-19a promoter between the 96 bp/78 bp sites. To validate if STAT3 is the core element regulating miR-19a transcription, a ChIP-qPCR assay was performed. An enrichment of STAT3 fragments was confirmed at the 96 bp/78 bp sites on the miR-19a promoter (Figure 3(d)). After that, the expression of STAT3 in the collected tissues was examined. It was found that the STAT3 expression was significantly increased in the tumor tissues compared to the adjacent ones and in the hypoxia-induced CRC cells (Figure 3(e)), which showed a positive correlation with miR-19a expression (Figure 3(f)). In addition, the western blot analysis confirmed an increase in the phosphorylation of STAT3 in CRC cells after hypoxia exposure (Figure 3(g)). These results, collectively, indicated that STAT3 possibly mediates miR-19a expression to involve in the progression of CRC.

### 3.4. miR-19a Mediates the PI3K/AKT Signaling Pathway

**Figure 4:**
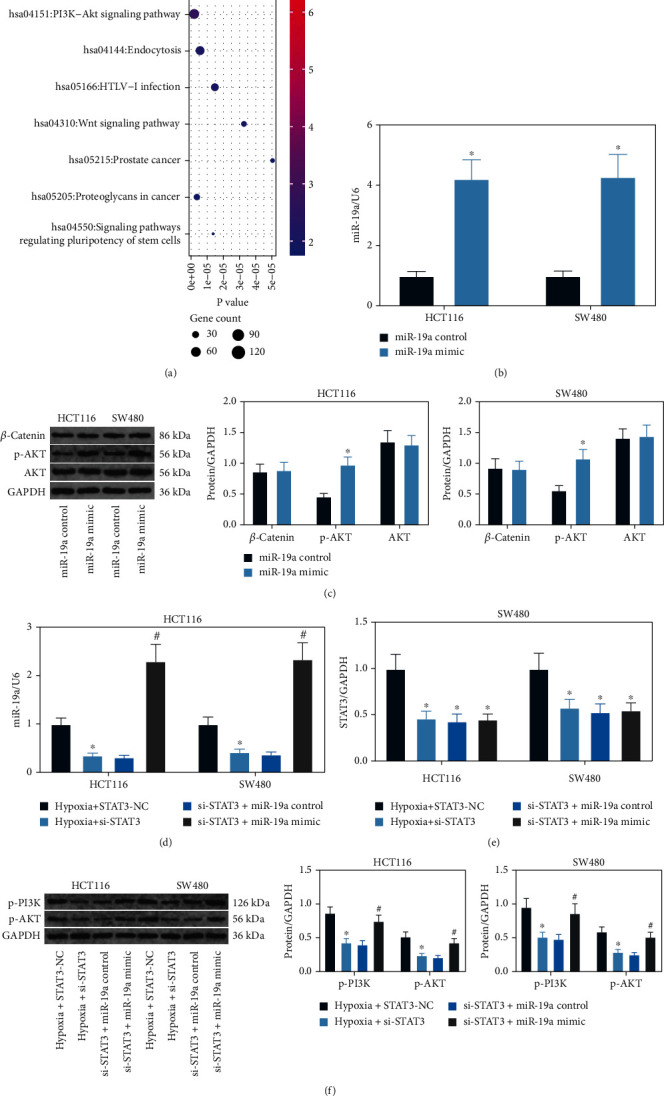
miR-19a mediates the PI3K/AKT signaling pathway in the hypoxia-exposed CRC cells: (a) downstream mRNAs of miR-19a predicted using the StarBase system; (b) transfection efficacy of miR-19a mimic examined using RT-qPCR (^∗^*p* < 0.05, two-way ANOVA); (c) activation of PI3K/AKT and Wnt/*β*-catenin signaling pathways in HCT116 and SW480 cells after miR-19a mimic transfection examined by western blot analysis (^∗^*p* < 0.05, two-way ANOVA); (d, e) expression of miR-19a and STAT3 in HCT116 and SW480 cells after si-STAT3 or si-STAT3+miR-19a mimic transfection examined by RT-qPCR (^∗#^*p* < 0.05, two-way ANOVA; ^∗^compared to hypoxia+STAT3-NC; ^#^compared to si-STAT3+miR-19a control group); (f) phosphorylation of PI3K and AKT in cells examined by western blot analysis (^∗#^*p* < 0.05, two-way ANOVA; ^∗^compared to hypoxia+STAT3-NC; ^#^compared to si-STAT3+miR-19a control group). Data were presented as the mean ± SD from three independent experiments.

To explore the downstream molecules, we predicted the target mRNAs of miR-19a using a bioinformatic system StarBase (http://starbase.sysu.edu.cn/). Thereafter, a Kyoto Encyclopedia of Genes and Genomes (KEGG) enrichment analysis was performed, which suggested that most of the target genes were enriched in the PI3K/AKT and Wnt/*β*-catenin signaling pathways (Figure 4(a)). To examine the signaling pathway implicated, miR-19a mimic was introduced into HCT116 and SW480 cells, and the successful upregulation was confirmed by RT-qPCR (Figure 4(b)). The activation of PI3K/AKT and Wnt/*β*-catenin signaling pathways in cells was examined by western blot analysis. Importantly, it was found that the phosphorylation of AKT was increased in cells after miR-19a overexpression, but the activation of the Wnt/*β*-catenin signaling pathway was not significantly changed (Figure 4(c)). In the subsequent experiments, si-STAT3 or si-STAT3 + miR-19a mimic was transfected into hypoxia-exposed cells. The successful transfections were confirmed by RT-qPCR again, and si-STAT3 was found to suppress expression of miR-19a (Figures 4(d) and 4(e)). The activation of the PI3K/AKT signaling pathway was examined by western blot analysis. It was found that the phosphorylation of PI3K and AKT was reduced after STAT3 knockdown but then restored following further miR-19a overexpression (Figure 4(f)). These results indicated that miR-19a activates the PI3K/AKT signaling pathway in CRC cells.

### 3.5. miR-19a Directly Targets PTEN mRNA

**Figure 5:**
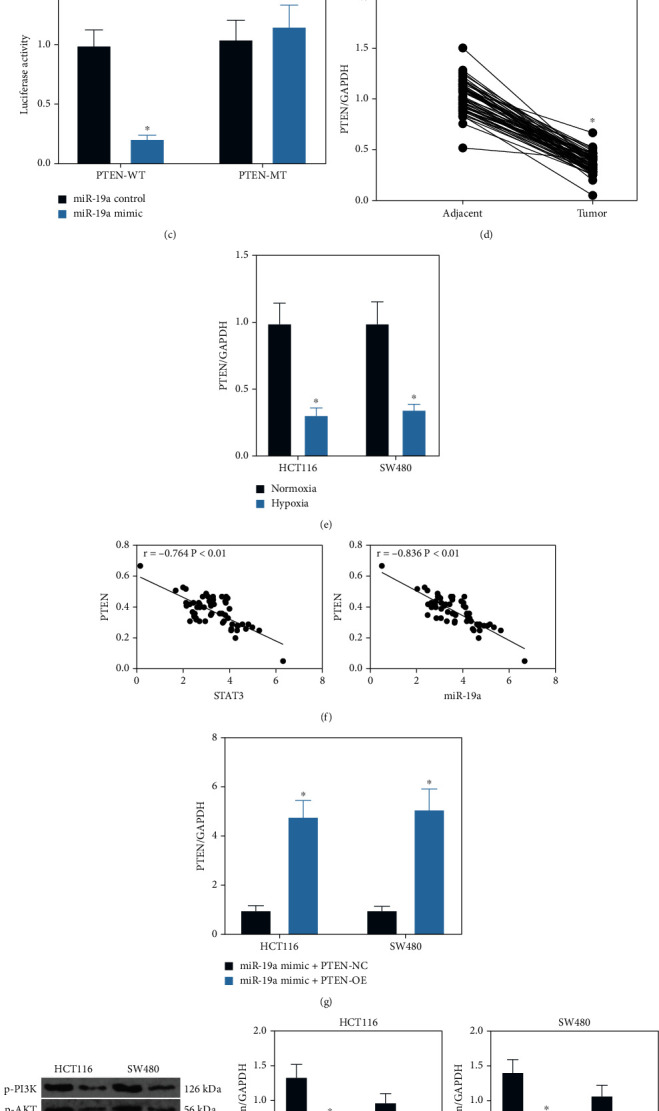
miR-19a directly targets PTEN mRNA in the hypoxia-exposed CRC cells: (a) a Venn diagram for the intersections of target mRNAs of miR-19a predicted using three bioinformatic systems and the mRNAs enriched in the PI3K/AKT signaling pathway; (b) expression of six candidate mRNAs in cells overexpressing miR-19a examined by RT-qPCR (^∗^*p* < 0.05, two-way ANOVA); (c) binding relationship between miR-19a and PTEN mRNA validated using a luciferase assay (^∗^*p* < 0.05, two-way ANOVA); (d) PTEN expression in the collected CRC tumor tissues and the adjacent tissues detected by RT-qPCR (^∗^*p* < 0.05, paired *t*-test); (e) PTEN expression in cells cultured under hypoxic conditions examined by RT-qPCR (^∗^*p* < 0.05, two-way ANOVA); (f) negative correlations between PTEN and STAT3/miR-19a in the CRC tissues (^∗^*p* < 0.05, Pearson's correlation analysis); (g) miR-19a and PTEN mRNA expression in cells after miR-19a mimic and PTEN-OE transfections examined by RT-qPCR (^∗^*p* < 0.05, two-way ANOVA); (h) phosphorylation of PI3K and AKT in cells detected by the western blot analysis (^∗^*p* < 0.05, two-way ANOVA). Data were presented as the mean ± SD from three independent experiments.

Following the findings above, we further predicted the target mRNAs of miR-19a using several bioinformatic systems including TargetScan (http://www.targetscan.org/vert_72/), RNA22 (https://cm.jefferson.edu/rna22/) and miRbase (http://mirbase.org/index.shtml). The common targets were further compared with the genes identified above which were enriched in the PI3K/AKT signaling pathway, and six genes were suggested to be intersected (Figure 5(a)). Among the six candidate genes, only PTEN was notably downregulated in the HCT116 cells overexpressing miR-19a (Figure 5(b)). Then, the binding relationship between miR-19a and PTEN was validated through a dual-luciferase reporter gene assay. Importantly, co-transfection with miR-19a mimic significantly reduced the luciferase activity of PTEN-WT vector in cells, while the luciferase activity in cells with other transfections was not changed (Figure 5(c)). The expression of PTEN in the collected tissues was then examined. It was found that PTEN was downregulated in the cancer tissues compared to the paracancerous tissues from the CRC patients (Figure 5(d)). In addition, the PTEN expression was downregulated in tissues cultured under hypoxic conditions (Figure 5(e)). Significant negative correlations were identified between PTEN and STAT3/miR-19a expression in the CRC tissues (Figure 5(f)). Subsequently, miR-19a mimic+PTEN-OE or miR-19a mimic + PTEN-NC were administrated into HCT116 and SW480 cells for further experiments. The successful transfections were confirmed by RT-qPCR (Figure 5(g)). The following western blot analysis results suggested that the phosphorylation of PI3K and AKT in cells increased by miR-19a mimic was blocked after further PTEN upregulation (Figure 5(h)).

### 3.6. Hypoxia Induces the STAT3/miR-19a/PTEN Axis to Affect Activity of the CRC Cells

**Figure 6:**
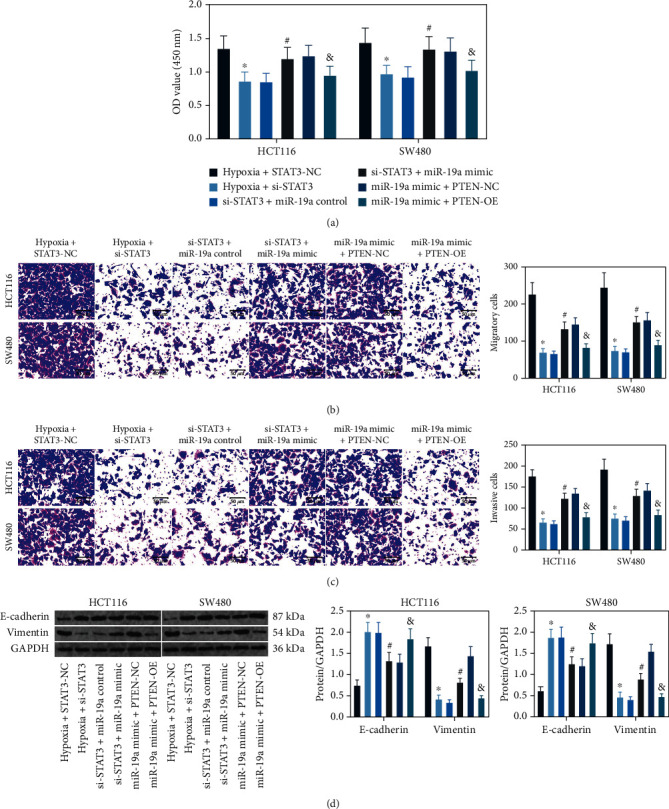
Hypoxia induces the STAT3/miR-19a/PTEN axis to affect activity of the CRC cells: (a) proliferation and viability of cells examined by the CCK-8 assay (^∗^^#&^*p* < 0.05, two-way ANOVA); (b, c) migration (b) and invasion (c) of cells examined by Transwell assays (^∗^^#&^*p* < 0.05, two-way ANOVA); (d) protein levels of E-cadherin and Vimentin in HCT116 and SW480 cells examined by western blot analysis (^∗^^#&^*p* < 0.05, two-way ANOVA). Data were presented as the mean ± SD from three independent experiments. ^∗^Compared to the hypoxia + STAT3-NC group; ^#^compared to the si-STAT3 + miR-19a control group; ^&^compared to the miR-19a mimic + PTEN-NC group.

After above transfections, the viability of cells was examined using the CCK-8 method. It was found that the viability of cells increased by hypoxia exposure significantly declined after STAT3 silencing but then restored after miR-19a upregulation. However, further overexpression of PTEN blocked the functions of miR-19a mimic on HC5-116 and SW480 cells (Figure 6(a)). Similar trends were found in cell migration and invasion. The number of cells that migrated and invaded into the lower membranes was decreased after si-STAT3 transfection but increased after further miR-19a mimic administration. Still, overexpression of PTEN blocked the migration and invasion of cells (Figures 6(b) and 6(c)). Moreover, the EMT-related markers in cells were examined using western blot analysis. Either si-STAT3 or PTEN-OE transfection notably reduced the expression of Vimentin but increased the expression of E-cadherin, but miR-19a mimic increased the ratio of E-cadherin to Vimentin, indicating that STAT3/miR-19a suppresses PTEN expression and promotes EMT of cells (Figure 6(d)).

### 3.7. The STAT3/miR-19a/PTEN Axis Affects CRC Tumorigenesis In Vivo

**Figure 7:**
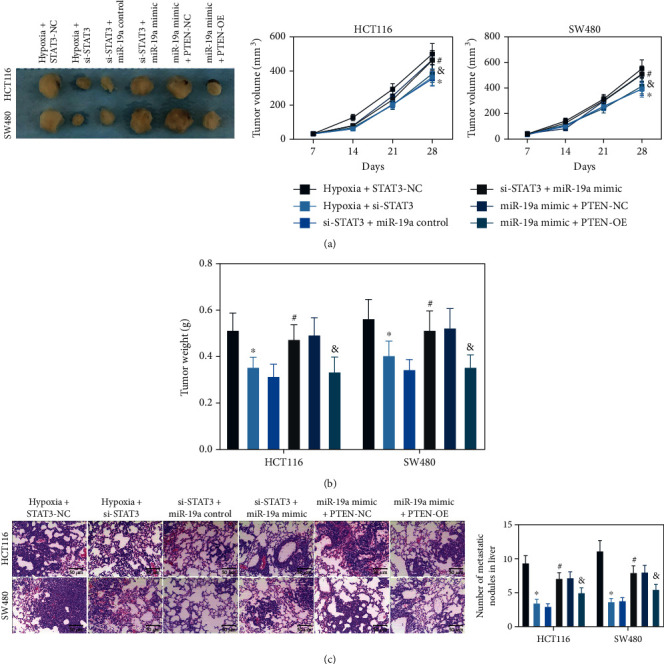
The STAT3/miR-19a/PTEN axis affects CRC malignancy *in vivo*: (a) volume of the xenograft tumors in nude mice (^∗^^#&^*p* < 0.05, two-way ANOVA); (b) weight of the xenograft tumors in nude mice (^∗^^#&^*p* < 0.05, two-way ANOVA); (c) number of metastatic nodules in murine liver tissues examined by HE staining (^∗^^#&^*p* < 0.05, two-way ANOVA). *N* = 5 in each group. ^∗^Compared to the hypoxia + STAT3-NC group; ^#^compared to the si-STAT3 + miR-19a control group; ^&^compared to miR-19a mimic + PTEN-NC group.

The hypoxia-exposed CRC cells were stably transfected with si-STAT3, miR-19a mimic, or PTEN-OE and then injected into the ventral side of mice. The tumor volume was evaluated once a week. It was found that downregulation of STAT3 or overexpression of PTEN reduced the volume of xenograft tumors in cells, whereas upregulation of miR-19a reduced the volume of tumors in mice (Figure 7(a)). On the 28^th^ day, the mice were euthanized, and the tumor weight was examined. Likewise, si-STAT3 or PTEN-OE transfections in cells reduced the weight of xenograft tumors in nude mice, whereas overexpression of miR-19a increased the weight of tumors (Figure 7(b)). In addition, CRC cells were further injected into mice through the tail vein for metastasis measurement. It was found that the number of metastatic nodules in murine liver tissues was significantly reduced after STAT3 knockdown or PTEN overexpression, whereas miR-19a mimic increased the tumor metastasis *in vivo* (Figure 7(c)).

## 4. Discussion

Hypoxia microenvironment is beneficiary for cancer cell proliferation, migration, and aggressiveness and the development of drug resistance and treating failure [6]. Identifying critical molecular mechanisms involved in hypoxia-induced cancer development is of potential for CRC control. In this research, we reported that hypoxia exposure induces activation of STAT3, which promotes miR-19a transcription and the subsequent inhibition of PTEN, thus triggering malignant behaviors of CRC.

The initial finding of this study was that hypoxia exposure increased proliferation, migration, invasion, and EMT activity of two CRC cell lines HCT116 and SW480. Cancer cells can adapt the signaling pathways which regulate proliferation, angiogenesis, and death, allowing tumors grow even under hypoxic conditions [14]. The role of hypoxia in tumor development, particularly in drug resistance, has been witnessed in an array of neoplastic cells [15–17]. This is also true for CRC, in which hypoxia has been revealed to induce drug resistance and growth of CRC cells through a HIF-1*α*/miR-338-5p/IL-6 axis [18].

miRNAs, a class of master regulators in cancer development, are frequently involved in the mediation by hypoxia. For instance, miR-210 has been reported as a robust hypoxia-regulated miRNA which is upregulated after HIF activation in cancers [19, 20]. In the present study, a miRNA microarray analysis was performed, which confirmed that miR-19a was significantly upregulated in both HCT116 and SW480 cells after 48 h of hypoxia exposure. In a previous study by Hu et al., miR-19a-3p was identified as one of the most significantly upregulated miRNAs in hepatocellular carcinoma cells cultured under hypoxic conditions [21]. Upregulation of miR-19a has been analyzed as an independent risk of poor prognosis in multiple human malignancies [22]. In agreement with this, miR-19a has been found to be upregulated and play oncogenic roles in several cancers [23, 24], including CRC [25, 26]. Hence, miR-19a might be an important effector of the hypoxia-mediated CRC development.

However, the mechanism responsible for the upregulation of miR-19a after hypoxia was unclear. We then focused on the upstream transcription factors of this miRNA. Importantly, the luciferase reporter gene assay suggested that transcription of miR-19a was highly activated within the 100 bp site on the promoter, and then, STAT3 was confirmed to be important for miR-19a transcription since it bound to miR-19a promoter at 96 bp/78 bp sites. Emerging studies have suggested that STAT3 is closely correlated with hypoxia. For instance, hypoxia-induced exosomes enhanced aggressiveness and chemoresistance of ovarian cancer cells through the activation of STAT3 [27]. Culture under hypoxic conditions has been observed to elevate expression of HIF-1*α* and STAT3 in oesophageal squamous cell carcinoma cells, leading to activated EMT [28]. Likewise, hypoxia activated the HIF-1*α*/STAT3 axis and promote invasion and EMT of CRC cells [29]. In this study, altered expression of STAT3 and miR-19a was introduced into the hypoxia-exposed cells. Importantly, the hypoxia-induced cell proliferation, migration, invasion, and EMT activity were significantly reduced upon STAT3 silencing while restored after miR-19a upregulation. Similar trends were found *in vivo* since si-STAT3 reduced growth and metastasis of xenograft tumors in nude mice, and the growth of tumors was re-enhanced by miR-19a mimic. These results validated that the STAT3/miR-19a axis is accountable for the hypoxia-induced CRC aggressiveness.

Following the findings above, the target mRNAs of miR-19a were predicted, which were analyzed to be enriched in the PI3K/AKT signaling pathway. This axis regulates an extended array of cellular behaviors, including proliferation, growth, cell size, metabolism, and motility [30]. Activation of the PI3K/Akt/mTOR signaling is essential for the development of various forms of cancers, especially in CRC, leaving this pathway as an important target for CRC treatment [31]. Here, we confirmed that miR-19a overexpression in CRC cells significantly activated the PI3K/AKT signaling pathway. The subsequent integrated analyses using three bioinformatic systems and cellular experiments confirmed that PTEN was the only target mRNA of miR-19a in the CRC cells. This may explain why the PI3K/AKT pathway was activated by miR-19a. PTEN is a widely accepted inhibitor of the PI3K/AKT/mTOR signaling pathway, and miRNAs that regulate PTEN are suggested as potential biomarkers indicating poor prognosis in CRC [32]. Quite in agreement with our findings, miR-19a has been found as a negative regulator of PTEN in ovarian cancer cells and promoted cell growth [33]. Interestingly, miR-19a has been reported to be responsible for hypoxia-induced proliferation and migration of human pulmonary arterial smooth muscle, during which the inhibition of PTEN was involved as well [34]. Here in this study, the malignant behaviors of CRC cells *in vitro* as well as the tumor growth and metastasis *in vivo*, which were encouraged by miR-19a mimic, were blocked following further PTEN upregulation. However, due to the time and funding limitations, the hypoxia condition in the tumor microenvironment in the animal experiments was not examined. We would like to focus on this issue in our future experiments. Nevertheless, the current evidence demonstrated that the STAT3/miR-19a/PTEN axis can affect CRC tumorigenesis *in vivo*.

## 5. Conclusion

Collectively, these results indicated that the STAT3/miR-19a axis is activated upon hypoxia and promotes progression of CRC through the inhibition of PTEN and the subsequent activation of the PI3K/AKT pathway. STAT3 and miR-19a may serve as candidate targets for CRC management. We hope more studies will be launched to validate our findings and to provide new thoughts into the pathogenesis of CRC.

## Figures and Tables

**Table 1:** Primer sequences for RT-qPCR.

Gene	Primer sequence (5′-3′)
miR-19a	F: CTGGAGTGTGCAAATCTATGC
	R: GTGCAGGGTCCGAGGT

U6	F: GCTTCGGCAGCACATATACTAAAAT
	R: CGCTTCACGAATTTGCGTGTCAT

STAT3	F: CATCCTGAAGCTGACCCAGG
	R: TATTGCTGCAGGTCGTTGGT

PTEN	F: ACCAGGACCAGAGGAAACCT
	R: GCTAGCCTCTGGATTTGACG

GAPDH	F: CTGACTTCAACAGCGACACC
	R: GTGGTCCAGGGGTCTTACTC

RT-qPCR: reverse transcription quantitative polymerase chain reaction; miR-19a: microRNA-19a; STAT3: signal transducer and activator of transcription 3; PTEN: phosphatase and tensin homolog; GAPDH: glyceraldehyde-3-phosphate dehydrogenase; F: forward; R: reverse.

## Data Availability

The data used to support the findings of this study are included within the article.
